# Lnc RNA HOTAIR functions as a competing endogenous RNA to regulate HER2 expression by sponging miR-331-3p in gastric cancer

**DOI:** 10.1186/1476-4598-13-92

**Published:** 2014-04-28

**Authors:** Xiang-hua Liu, Ming Sun, Feng-qi Nie, Ying-bin Ge, Er-bao Zhang, Dan-dan Yin, Rong Kong, Rui Xia, Kai-hua Lu, Jin-hai Li, Wei De, Ke-ming Wang, Zhao-xia Wang

**Affiliations:** 1 Department of Biochemistry and Molecular Biology, Nanjing Medical University, Nanjing, People’s Republic of China; 2 Department of Oncology, First Affiliated Hospital, Nanjing Medical University, Nanjing, People’s Republic of China; 3 Department of Physiology, Nanjing Medical University, Nanjing, People’s Republic of China; 4 Department of General Surgery, First Affiliated Hospital, Nanjing Medical University, Nanjing, People’s Republic of China; 5 Department of Oncology, Second Affiliated Hospital, Nanjing Medical University, Nanjing, People’s Republic of China

**Keywords:** Competing endogenous RNA, HER2, HOTAIR, Gastric cancer, Proliferation and invasion

## Abstract

**Background:**

Accumulating evidence indicates that the long non-coding RNA HOTAIR plays a critical role in cancer progression and metastasis. However, the overall biological role and clinical significance of HOTAIR in gastric carcinogenesis remains largely unknown.

**Methods:**

HOTAIR expression was measured in 78 paired cancerous and noncancerous tissue samples by real-time PCR. The effects of HOTAIR on gastric cancer cells were studied by overexpression and RNA interference approaches in vitro and in vivo. Insights of the mechanism of competitive endogenous RNAs (ceRNAs) were gained from bioinformatic analysis, luciferase assays and RNA binding protein immunoprecipitation (RIP). The positive HOTAIR/HER2 interaction was identified and verified by immunohistochemistry assay and bivariate correlation analysis.

**Results:**

HOTAIR upregulation was associated with larger tumor size, advanced pathological stage and extensive metastasis, and also correlated with shorter overall survival of gastric cancer patients. Furthermore, HOTAIR overexpression promoted the proliferation, migration and invasion of gastric carcinoma cells, while HOTAIR depletion inhibited both cell invasion and cell viability, and induced growth arrest in vitro and in vivo. In particular, HOTAIR may act as a ceRNA, effectively becoming a sink for miR-331-3p, thereby modulating the derepression of HER2 and imposing an additional level of post-transcriptional regulation. Finally, the positive HOTAIR/HER2 correlation was significantly associated with advanced gastric cancers.

**Conclusions:**

HOTAIR overexpression represents a biomarker of poor prognosis in gastric cancer, and may confer malignant phenotype to tumor cells. The ceRNA regulatory network involving HOTAIR and the positive interaction between HOTAIR and HER2 may contribute to a better understanding of gastric cancer pathogenesis and facilitate the development of lncRNA-directed diagnostics and therapeutics against this disease.

## Background

Gastric cancer is the second leading cause of cancer-induced death, and is the most common gastrointestinal malignancy in East Asia, Eastern Europe, and parts of Central and South America. In most patients, gastric cancer is diagnosed at an advanced stage and is accompanied by malignant proliferation, extensive invasion and lymphatic metastasis. Successful therapeutic strategies are limited and the mortality is high [1,2]. Long non-coding RNAs (lncRNAs) have recently gained significant attention in delineating the complex mechanisms underlying malignant processes such as carcinogenesis, metastasis and drug resistance. Therefore, if we want to fully understand gastric carcinogenesis, we need to consider this family of regulatory transcripts that add a new layer of complexity to tumor biology.

Although only a small number of functional lncRNAs have been well characterized to date, they have been shown to regulate gene expression at various levels, including chromatin modification, transcription and post-transcriptional processing [3,4]. Recently, a new regulatory mechanism has been identified in which crosstalk between lncRNAs and mRNAs occurs by competing for shared microRNAs (miRNAs) response elements. In this case, lncRNAs may function as competing endogenous RNAs (ceRNAs) to sponge miRNAs, thereby modulating the derepression of miRNA targets and imposing an additional level of post-transcriptional regulation [5]. In previous reports, a muscle-specific lncRNA, linc-MD1, has been reported to be a ceRNA that protects MyoD messenger RNA (mRNA) from miRNA-mediated degradation [6]. Pluripotency-associated lnc-RoR may function as a key ceRNA to link the network of miRNAs and core transcription factors, e.g., Oct4, Sox2, and Nanog, in human embryonic stem cells. Notably, lncRNA HULC is highly upregulated in liver cancer and plays an important role in tumorigenesis [7]. In particular, HULC may act as an endogenous ‘sponge’ that down-regulates a series of miRNAs activities, including miR-372 [8]. We therefore propose that some lncRNAs may also have roles as ceRNAs, linking miRNAs and the post-transcriptional network in gastric pathogenesis.

HOTAIR (*Hox transcript antisense intergenic RNA*) is a ~2.2-kb long non-coding RNA transcribed from the *HOXC* locus, which can repress transcription *in trans* of *HOXD* in foreskin fibroblasts [9]. As a novel molecule in the field of tumor biology, HOTAIR initially became well known for its involvement in primary breast tumors and breast cancer metastases, wherein elevation of HOTAIR promoted invasiveness and metastasis [10]. Furthermore, HOTAIR expression positively correlates with malignant processes and poor outcome in colorectal cancer, hepatocellular carcinoma, pancreatic cancer and gastrointestinal stromal tumors [11-14]. Recent studies reported that HOTAIR was upregulated in gastric cancer [15,16]. Nevertheless, the overall biological role and underlying molecular mechanism of HOTAIR in gastric carcinogenesis remains largely undefined.

In this study, we report that HOTAIR upregulation is a characteristic molecular change in gastric cancer and investigate the biological roles of HOTAIR on the phenotypes of gastric cancer cells in vitro and in vivo. Moreover, mechanistic analysis reveals that HOTAIR may function as a ceRNA to regulate the expression of human epithelial growth factor receptor 2 (HER2) through competition for miR-331-3p, thus playing an oncogenic role in gastric pathogenesis. The present work provides the first evidence for a positive HOTAIR/HER2 correlation and the crosstalk between miR-331-3p, HOTAIR and HER2, shedding new light on the treatment of gastric cancer.

## Results

### HOTAIR expression is upregulated in human gastric cancer tissues

**Figure 1:**
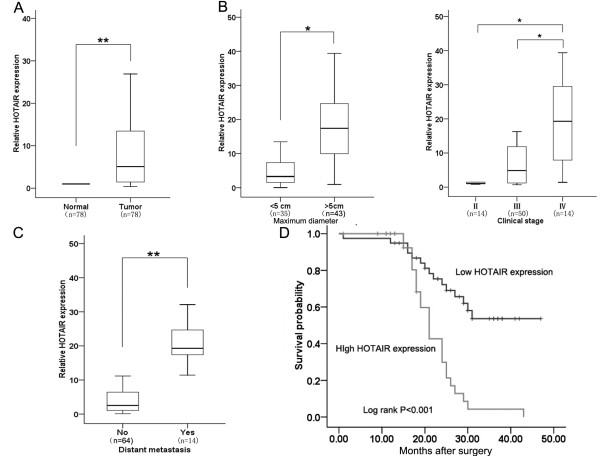
**Relative HOTAIR expression in gastric cancer tissues and its clinical significance. (A)** Relative expression of HOTAIR in gastric cancer tissues (n = 78) in comparison with corresponding non-tumor normal tissues (n = 78). HOTAIR expression was examined by qRT-PCR and normalized to GAPDH expression. Data was presented as fold-change in tumor tissues relative to normal tissues. **(B)** Examination of the correlation between HOTAIR expression and clinical pathological features showed that HOTAIR upregulation correlated with larger tumor size and advanced pathological stage. **(C)** HOTAIR expression was significantly higher in patients with distant metastasis than in those with non-distant metastasis. **(D)** Kaplan–Meier overall survival curves according to HOTAIR expression level. The overall survival of the High-HOTAIR group (n = 39: HOTAIR expression ratio ≥ median ratio) was significantly higher than that of Low-HOTAIR group (n = 39; HOTAIR expression ratio ≤ median ratio; P < 0.001, log-rank test). *P < 0.05, **P < 0.01.

The level of HOTAIR expression was determined in 78 paired gastric cancer samples and adjacent, histologically normal tissues by qRT-PCR, and normalized to GAPDH. HOTAIR expression was significantly upregulated in cancerous tissues (mean ratio of 14.35-fold, P < 0.01) compared with normal counterparts (Figure 1A). Examination of the correlation between HOTAIR expression and clinical pathological features showed that HOTAIR upregulation was correlated with larger tumor size, advanced pathological stage, distant metastasis (Figure 1B and C), lymph node metastasis and tumor cell differentiation (Table 1). However, HOTAIR expression was not associated with tumor position or patient gender (Table 1). With regard to overall survival, patients with high HOTAIR expression had a significantly poorer prognosis than those with low HOTAIR expression (P < 0.001, log-rank test; Figure 1D). These results imply that HOTAIR overexpression may be useful in the development of novel prognostic or progression markers for gastric cancer.

**Table 1:** Correlation of the expression of HOTAIR with clinicopathologic features

**Clinicopathologic features**	**n (%)**	**Relative expression of HOTAIR**^**a**^	**P-value**^**b**^
Gender			P = 0.62
Male	50 (64)	12.21 (0.97-17.53)	
Female	28 (36)	15.01 (7.34 -19.52)	
Site of tumor			P = 0.910
Distal third	18 (23)	9.1 (1.37-14.12)	
Middle third	20 (26)	7.96 (2.55-9.72)	
Proximal stomach	40 (51)	7.76 (1.24-13.58)	
Differentiation			P = 0.045
Poor	56 (72)	15.6 (6.25-20.36)	
High/moderate	22 (28)	8.1 (0.98-11.14)	
Lymph node Metastasis			P = 0.005
N0	10 (13)	1.14 (0.75-1.42)	
N1	11 (14)	3.44 (1.15-4.85)	
N2	29 (37)	11.99 (7.15-14.77)	
N3	28 (36)	32.58 (7.34-36.27)	
Metastatic disease			P = 0.029
M0	64 (82)	9.35 (2.53-33.64)	
M1	14 fig	28.82 (16.39-39.39)	

^a^Median of relative expression, with 25th-75th percentile in parentheses. ^b^P < 0.05 was considered significant (Mann–Whitney *U* test between 2 groups and Kruskall-Wallis test for 3 groups).

### Manipulation of HOTAIR levels in gastric cancer cells

**Figure 2:**
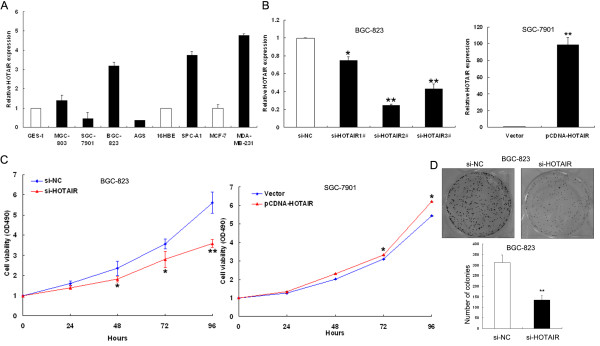
**The level of HOTAIR expression in gastric cancer cells and its effect on cell proliferation in vitro. (A)** Results from qRT-PCR demonstrating HOTAIR expression levels of gastric cancer cell lines (MGC-803, SGC-7901, BGC-823, and AGS) compared with normal gastric epithelium cell line (GES-1), NSCLC cell line (SPC-A1) compared with normal human bronchial epithelial cell line (16HBE), and breast cancer cell lines MDA-MB-231 compared with MCF-7. **(B)** qRT-PCR analyses of HOTAIR expression level following treatment BGC-823 cells with siRNAs targeting HOTAIR and treatment SGC-7901 cells with pCDNA/HOTAIR vector. **(C)** MTT assay was performed to determine the proliferation of si-HOTAIR-transfected BGC-823 cells or pCDNA/HOTAIR-transfected SGC-7901 cells. Data represent the mean ± s.d. from three independent experiments. **(D)** Colony-forming growth assay was performed to determine the proliferation of si-HOTAIR transfected BGC-823 cells. Colonies were counted and captured. *P < 0.05 and **P < 0.01.

We next performed qRT-PCR analysis to examine the expression levels of HOTAIR in various cancer cell lines, including gastric, non-small cell lung cancer (NSCLC) and breast cancer-derived cells. As shown in Figure 2A, of the four gastric cancer cell lines investigated (MGC-803, SGC-7901, BGC-823, and AGS), BGC-823 expressed higher levels of HOTAIR (3.18-fold) than the normal gastric epithelium cell line (GES-1). Similarly, NSCLC cell line, SPC-A1, showed a 3.73-fold upregulation of HOTAIR over the normal human bronchial epithelial cell line, 16HBE. The highly metastatic breast cancer cell line MDA-MB-231 showed a 4.77-fold upregulation of HOTAIR compared with MCF-7 (Figure 2A). Our data suggest that HOTAIR may be frequently upregulated in many tumor cells.

To manipulate HOTAIR levels in gastric cancer cells, a pCDNA/HOTAIR vector was transfected into SGC-7901 cells and si-HOTAIR was transfected into BGC-823 cells, respectively. qRT-PCR analysis of HOTAIR levels was performed at 48 h post-transfection and revealed that HOTAIR expression was increased 98-fold in SGC-7901 cells, compared with control cells. However, in BGC-823 cells, HOTAIR expression was effectively 75% knocked down by si-HOTAIR2, the most effective siRNA subsequently used in the following experiments (Figure 2B).

### Effect of HOTAIR on cell proliferation and apoptosis in vitro

The significant increase of HOTAIR expression in gastric cancer samples prompted us to explore the possible biological significance of HOTAIR in tumorigenesis. MTT assay revealed that cell growth was significantly impaired in BGC-823 cells transfected with si-HOTAIR, while proliferation of SGC-7901 cells was increased in pCDNA/HOTAIR transfected cells compared with respective controls (Figure 2C). Similarly, the result of colony-formation assay revealed that clonogenic survival was decreased following inhibition of HOTAIR in BGC-823 cells (Figure 2D).

**Figure 3:**
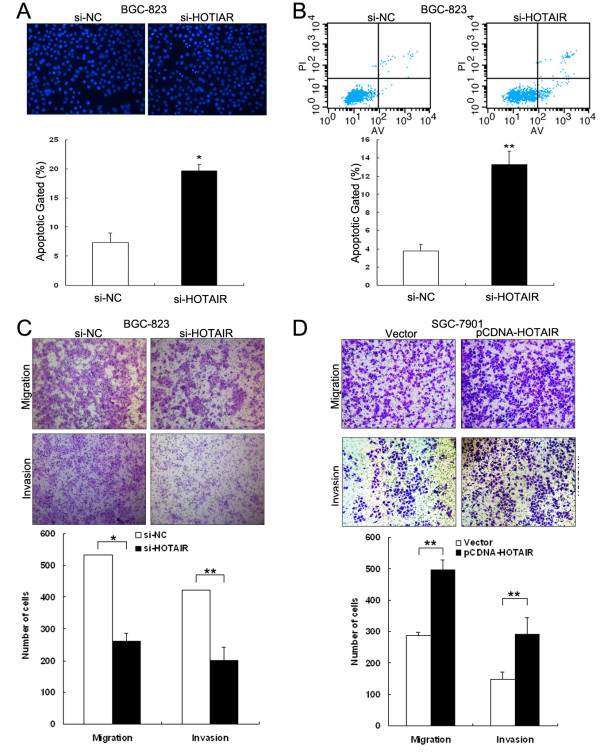
**The effect of HOTAIR on gastric cancer cell apoptosis, migration and invasion *in vitro*.** BGC-823 cells were transfected with si-HOTAIR or si-NC, and SGC-7901 cells were transfected with pCDNA/HOTAIR vector or empty vector control. **(A)** Hoechst staining assay of cell apoptosis; the percentage of Hoechst-positive nuclei per optical field (at least 50 fields) was determined. **(B)** The apoptotic rates of cells were detected by flow cytometry. UL, necrotic cells; UR, terminal apoptotic cells; LR, early apoptotic cells. **(C and D)** Transwell assays were performed to investigate changes in cell migration and invasiveness. *P < 0.05 and **P < 0.01.

To determine whether apoptosis was a contributing factor to cell growth inhibition, we performed Hoechst staining and flow-cytometric analysis of si-HOTAIR-treated BGC-823 cells. The data showed that the number of cells with condensed and fragmented nuclei indicating the fraction of early apoptotic cells was significantly higher in si-HOTAIR-treated BGC-823 cells compared with si-NC-treated cells (Figure 3A and B). In addition, we found that inhibition of HOTAIR enhanced caspase-3-dependent apoptosis, demonstrated by western blot analysis of activated caspase-3 after si-HOTAIR transfection (Additional file 1: Figure S1). Taken together, these results indicate that knockdown of HOTAIR suppresses gastric cancer cell proliferation and induces apoptosis in vitro.

### HOTAIR promotes migration and invasion of gastric cancer cells in vitro

Cell invasion is a significant aspect of cancer progression that involves the migration of tumor cells into contiguous tissues and the dissolution of extracellular matrix proteins. Here we evaluated cancer cell invasion through transwell assays. As shown in Figure 3C, the transfection of HOTAIR siRNA impeded the migratory ability BGC-823 cells by roughly 66%. A corresponding effect on invasiveness was also observed in a parallel invasion assay. Conversely, transfection of SGC-7901 cells with pCDNA/HOTAIR vector promoted cell migration and invasiveness ~1.9-fold (Figure 3D). These data indicate that HOTAIR has oncogenic properties that can promote a migratory and invasive phenotype in gastric cancer cells.

### HOTAIR promotes tumorigenesis of gastric cancer cells in vivo

**Figure 4:**
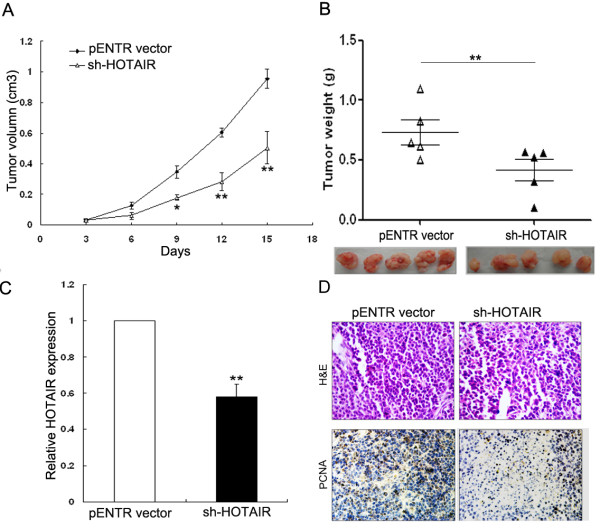
**Effect of HOTAIR knockdown on tumor growth *in vivo*. (A)** Tumor growth curves were measured after injection of BGC-823 cells transfected with sh-HOTAIR or pENTR vector. Tumor volume was calculated every 3 days. Data are presented as mean ± s.d. (n = 5). **(B)** Tumor weight. Values are means of tumor weight ± s.d. **(C)** qRT-PCR analysis of HOTAIR expression in tissues of resected tumors. **(D)** Tumors developed from BGC-823/sh-HOTAIR cells showed a lower level of PCNA protein than tumors developed from BGC-823/pENTR vector cells. Upper: H & E staining; Lower: immunostaining. *P < 0.05 and **P < 0.01.

To explore whether the level of HOTAIR expression affects tumorigenesis, BGC-823 cells transduced with the sh-HOTAIR/pENTR vector (EV) were used in a nude mice xenograft model. Up to 16 days after knockdown of HOTAIR, there was a dramatic decrease in tumor volume and weight in the sh-HOTAIR group compared with controls (Figure 4A, B and C). Next, immunostaining analysis of the proliferation marker PCNA was performed in resected tumor tissues. In comparison with that in tumors formed from control cells, sh-HOTAIR-derived tumors showed significantly reduced PCNA positivity (Figure 4D). These results suggest that the level of HOTAIR expression is significantly associated with the in vivo proliferation capacity of gastric cancer cells.

### HOTAIR is a target of miR-331-3P and miR-124

**Figure 5:**
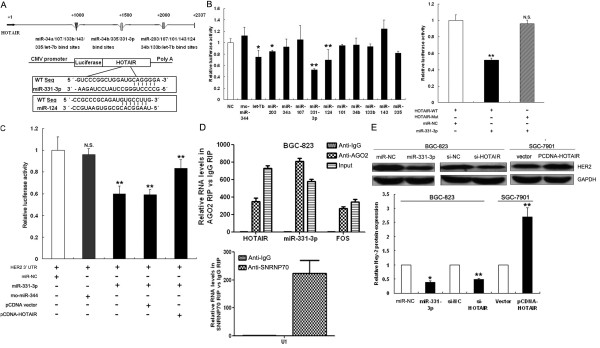
**HOTAIR is a target of miR-331-3P or miR-124 and controls miR-331-3p target. (A)** The 11 putative miRNA binding sites in the HOTAIR sequence. The HOTAIR cDNA containing the putative miRNAs recognition sites was cloned downstream of the luciferase gene and named RLuc-HOTAIR. **(B)** Left: The luciferase reporter plasmid (RLuc-HOTAIR) was co-transfected into HEK-293 T cells with the 11 various miRNA-coding plasmids. Right: the luciferase reporter plasmid containing wild-type or mutant HOTAIR was co-transfected into HEK-293 T cells with miR-331-3p in parallel with an empty plasmid vector. Luciferase activity was determined using the dual luciferase assay and shown as the relative luciferase activity normalized to renilla activity. Histogram indicates the values of luciferase measured 48 h after transfection. **(C)** The 3’-UTR of HER2 was fused to the luciferase coding region (RLuc-HER2 3’-UTR) and transfected in HEK293T cells with miR-331-3p to confirm HER2 is the target of miR-331-3p. RLuc-HER2 3’-UTR and miR-331-3p constructs were co-transfected into HEK293T cells with plasmids expressing HOTAIR (pCDNA/HOTAIR) or with a control vector to verify the ceRNA activity of HOTAIR. rno-miRNA-344 was used as a negative control. Histogram indicates the values of luciferase measured 48 h after transfection. **(D)** RIP with mouse monoclonal anti-Ago2, preimmune IgG or 10% input from BGC 823-cell extracts. RNA levels in immunoprecipitates were determined by qRT-PCR. Top: levels of HOTAIR, miRNA331-3P and FOS RNA were presented as fold enrichment in Ago2 relative to IgG immunoprecipitates. Bottom: relative RNA levels of U1 snRNA in SNRNP70 relative to IgG immunoprecipitates. Numbers are mean ± s.d. (n = 3). **(E)** Western blot analysis of HER2 protein level following treatment of BGC-823 cells with si-HOTAIR or pCDNA/HOTAIR, and SGC-7901 cells with pCDNA/HOTAIR. GAPDH was used as control. *P < 0.05, **P < 0.01 and N.S. not significant.

Bioinformatics analysis of miRNA recognition sequences on HOTAIR revealed the presence of 11 tumor-suppressive miRNAs binding sites. The HOTAIR cDNA was cloned downstream of the luciferase gene and named RLuc-HOTAIR (Figure 5A), then transfected together with various miRNA-coding plasmids. rno-miR-344 acted as a negative control. The results showed that luciferase activity was reduced by 48% and 31% compared with the empty vector control when miR-331-3p and miR-124 were expressed, respectively. These data demonstrate that both miR-331-3p and miR-124 can directly bind to HOTAIR through respective miRNA recognition sites (Figure 5B left panel).

Herein, we chose miR-331-3p as a model miRNA for further studies. To further confirm that the reduction in luciferase activity from the RLuc-HOTAIR-WT vector was due to direct interaction between the miRNA and its putative binding site, we mutated the miR-331-3p binding site by site-directed mutagenesis, resulting in RLuc-HOTAIR-Mut. As expected, suppression of luciferase activity was completely abolished in this mutant construct compared with wild-type vector (Figure 5B right panel).

### HOTAIR and miR-331-3p both bind with AGO2 in gastric cancer cells

miRNAs are known to be present in the cytoplasm in the form of miRNA ribonucleoprotein complexes (miRNPs) that also contain Ago2, the core component of the RNA-induced silencing complex (RISC) [17,18]. To test whether HOTAIR associates with miRNPs, RNA binding protein immunoprecipitation (RIP) experiments were performed on BGC-823 cell extracts using antibodies against Ago2. RNA levels in immunoprecipitates were determined by qRT-PCR. HOTAIR was preferentially enriched (354-fold) in Ago2-containing miRNPs relative to control immunoglobulin G (IgG) immunoprecipitates. Similarly, miRNA-331-3p was detected at a level 840-fold greater than that of control anti-IgG. Successful immunoprecipitation of Ago2-associated RNA was verified by qRT-PCR, using RIP primers against human FOS included in the RIPAb + Ago2 kit (Figure 5D, top panel). Moreover, anti-SNRNP70 was used as a positive control for the RIP procedure, and U1 snRNA was also detected at a level 206-fold greater than that of anti-IgG (Figure 5D, bottom panel). Thus, HOTAIR is present in Ago2-containing miRNPs, likely through association with miRNA-331-3p, consistent with our bioinformatic analysis and luciferase assays.

### HOTAIR controls the miR-331-3p target, HER2

Among the many targets of miR-331-3p, we concentrated on HER2 since it encodes a transmembrane protein with a relevant function in carcinogenesis and resistance to trastuzumab-based therapy [19]. The 3’-UTR of HER2 was fused to the luciferase coding region (RLuc-HER2 3’-UTR) and transfected with plasmids encoding miR-331-3p and an empty plasmid vector. rno-miRNA-344 acted as a negative control. The luciferase assay showed that miR-331-3p significantly inhibited luciferase activity (~41% inhibition) of the RLuc-HER2 3’-UTR reporter, confirming that HER2 is a target of miR-331-3p. The RLuc-HER2 3’-UTR construct was subsequently transfected together with plasmids encoding miR-331-3p and HOTAIR (pCDNA/HOTAIR). Luciferase assays indicated that, in the presence of HOTAIR, RLuc-HER2 3’-UTR repression was restored compared with the control group (Figure 5C). This indicates that HOTAIR acts as an endogenous ‘sponge’ by binding miR-331-3p, thus abolishing the miRNA-induced repressing activity on the HER2 3’-UTR.

Furthermore, the effect of HOTAIR expression on endogenous HER2 protein in combination with modulation of miRNA or lncRNA levels was monitored by the different approaches shown in Figure 5E: (1) miR-331-3p overexpression against HER2 in BGC-823 cells; (2) HOTAIR knockdown against HER2 in BGC-823 cells; (3) HOTAIR overexpression for HER2 in SGC-7901 cells. Western blot analysis showed that forced expression of miR-331-3p or knockdown of HOTAIR in BGC-823 cells triggered a significant silencing effect on endogenous HER2 protein expression. Furthermore, HER2 protein expression was markedly upregulated after transfection with HOTAIR in SGC-7901 cells, which demonstrates a relatively low endogenous HOTAIR expression level.

Together these data indicate that by binding miR-331-3p, HOTAIR acts as a ceRNA for the target HER2 mRNA, thereby modulating the derepression of HER2 and imposing an additional level of post-transcriptional regulation.

### MiR-331-3p and miR-124 suppress gastric cancer cells proliferation

**Figure 6:**
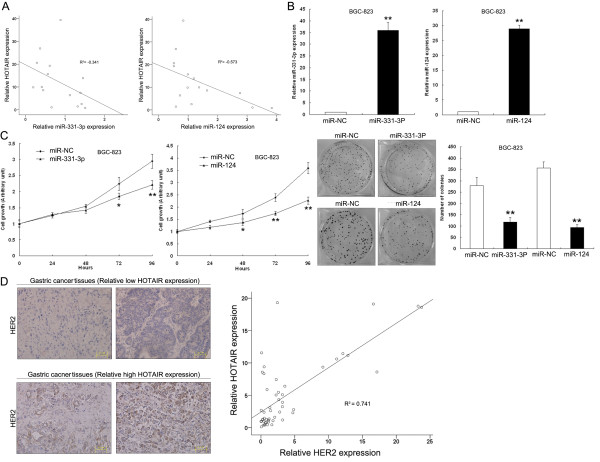
**Positive correlation between HOTAIR and HER2 expression and the relevance to miR-331-3p/miR-124 expression levels. (A)** Bivariate correlation analysis of the relationship between HOTAIR expression and miR-331-3p, or miR-124 level. **(B)** BGC-823 cells were transfected with plasmids encoding miR-331-3p or miR-124, and qRT-PCR was used to detect the miRNA levels compared with controls (miR-NC). **(C)** MTT assay and colony-forming growth assay were performed to determine the proliferation of miR-331-3p or miR-124 transfected BGC-823 cells. **(D)** Left: immunostaining of HER2 protein in advanced gastric cancer tissue samples. Upper: immunostaining of HER2 was negative in gastric cancer tissues with relatively low HOTAIR expression. Lower: immunostaining of HER2 was positive in gastric cancer tissues with relatively high HOTAIR expression. Right: bivariate correlation analysis of the relationship between HOTAIR and HER2 expression level. *P < 0.05, **P < 0.01.

To serve as an endogenous sink for target miRNAs, the abundance of HOTAIR should be comparable to or higher than miR-331-3p/miR-124. In our study, qRT-PCR analysis showed that miR-331-3p/miR-124 expression was inversely correlated with HOTAIR expression in 20 pairs of advanced gastric cancers (Figure 6A). To validate whether miR-331-3p and miR-124 could also inhibit gastric cancer cell proliferation, we forced their expression in BGC-823 cells using miRNA-encoding plasmids. The expression levels of miRNA-331-3p and miR-124 transfected into BGC-823 cells were significantly increased by 36-fold and 29-fold, respectively, compared with control cells (Figure 6B). Next, MTT and colony-formation assays were performed to determine cell viability. The results of the MTT assay and growth curves revealed that cells transfected with miR-331-3p or miR-124 showed significant growth retardation when compared with cells transfected with empty vector (Figure 6C). These data indicate that overexpression of miR-331-3p or miR-124 expression can arrest gastric cancer cell proliferation, which is consistent with the results of HOTAIR expression knockdown in BGC-823 cells.

### HER2 is coexpressed with HOTAIR in gastric cancer tissues

The HER2 overexpression rate was reported to be 7–34% in gastric cancer, and was associated with more aggressive disease and poorer survival in gastric cancer [19]. We detected expression of HER2 in 50 advanced gastric cancer tissues (stage III/IV) selected from the previous 78 gastric cancer tissues by immunohistochemistry (IHC) and qRT-PCR analysis. The results of IHC staining showed HER2 protein positivity in 56% of the selected 50 gastric cancer tissues. Eighty-six percent of these HER2 positive samples were from advanced stage III/IV tumors, and 71% displayed high HOTAIR expression (Figure 6D and Additional file 2: Table S2). Bivariate correlation analysis showed that expression of HER2 was significantly correlated with HOTAIR transcript level in gastric cancer tissues compared with normal counterparts (Figure 6D). These data indicate that the expression of HER2 is positively associated with upregulated HOTAIR in gastric cancer tissue samples, suggesting that characterization of the HER2/HOTAIR interaction might be biologically significant in human gastric tumorigenesis.

Notably, because of an ongoing phase III/IV trial, the HER2 overexpression rate detected in the present study is higher than the previously reported median range. This increased rate of HER2 overexpression is strongly linked to poor outcomes for patients with metastatic and high-grade localized gastric cancers, thereby highlighting the importance of HER2 in gastric cancer development and metastasis.

## Discussion

LncRNAs, which are more than 200 nucleotides in length with limited protein-coding capacity, are often expressed in a disease-, tissue- or developmental stage-specific manner, indicating specific functions for lncRNAs in development and diseases and making these molecules attractive therapeutic targets [20-22]. A number of recent papers have revealed that dysregulation of these lncRNAs may also affect the regulation of the eukaryotic genome and provide a cellular growth advantage, resulting in progressive and uncontrolled tumor growth [23-25]. Therefore, lncRNAs may provide a missing piece of the otherwise well-known oncogenic and tumor suppressor network puzzle.

In this study, we tested the expression of HOTAIR in gastric carcinoma samples and their surrounding non-tumorous tissues. We also identified the function of HOTAIR in gastric carcinoma cells by applying gain- and loss-of-function approaches. The results demonstrated that HOTAIR was upregulated in gastric carcinoma tissues in comparison with adjacent normal gastric tissues, and that HOTAIR upregulation correlated with larger tumor size, advanced pathological stage and extensive metastasis. Moreover, the overall survival time of patients with lower HOTAIR expression levels was significantly longer than that of patients with moderate or strong HOTAIR expression levels. Furthermore, HOTAIR overexpression promoted the proliferation, migration and invasion of gastric carcinoma cells, while HOTAIR depletion inhibited cell invasion and cell viability, and induced growth arrest both in vitro and in vivo. Additionally, HOTAIR suppression led to the promotion of gastric cell apoptosis. These findings suggest that HOTAIR plays a direct role in the modulation of multiple oncogenic properties and gastric cancer progression, stimulating new research directions and therapeutic options considering HOTAIR as a novel prognostic marker and therapeutic target in gastric cancer.

The importance of lncRNAs in human disease may be associated with their ability to impact cellular functions through various mechanisms. In this study, as far as the mechanism of HOTAIR is concerned, it is worth mentioning that subcellular localization analysis of HOTAIR by RNA fluorescence in situ hybridization assay demonstrates the localization of HOTAIR to both the nucleus and the cytoplasm [24]. It is evident that nuclear HOTAIR can target polycomb repressive complex 2, altering H3K27 methylation and gene expression patterns across the genome [10,11]. Recent work reported a scaffold function for HOTAIR in the cytoplasm as an inducer of ubiquitin-mediated proteolysis [26]. Nevertheless, the tumorigenic properties and mechanistic heterogeneity of HOTAIR, and particularly those of the cytoplasmic form, are far from being fully elucidated.

Inspired by the ‘competitive endogenous RNAs’ regulatory network and emerging evidence that suggests that lncRNAs may participate in this regulatory circuitry, we hypothesized that HOTAIR may also serve as a ceRNA and so we searched for potential interactions with miRNAs. In support of this notion, we employed bioinformatics analysis and luciferase assays to validate the direct binding ability of the predicted miRNA response elements on the full-length HOTAIR transcript. As expected, we discovered miR-331-3p and miR-124 could form complementary base pairing with HOTAIR and induce translational repression of a RLuc-HOTAIR reporter gene. In addition, HOTAIR:miR-331-3P coimmunoprecipitation with anti-Ago2 demonstrated a physical interaction in gastric cancer cells, providing further support for HOTAIR’s miRNA-sequestering activity. To serve as an endogenous ‘sponge’, the abundance of HOTAIR should be comparable to or higher than miR-331-3p/miR-124. In our study, qRT-PCR analysis showed that miR-331-3p/miR-124 expression was inversely correlated with HOTAIR expression in advanced gastric cancer. Moreover, ectopic overexpression of miR-331-3p or miR-124 expression could arrest gastric cancer proliferation, which was consistent with results of knockdown of HOTAIR expression in gastric cancer cells. Taken together, these data are consistent with our hypothesis and indicate that HOTAIR may interact with miRNAs to link miRNAs and the post-transcriptional network in gastric pathogenesis.

To investigate the miRNA-related functions of HOTAIR in gastric pathogenesis, we chose miR-331-3p as a model miRNA for further studies, with a particular focus on the target gene HER2. In carcinomas, HER2 acts as an oncogene, encoding a 185-kDa transmembrane protein to trigger the activation of cell signaling networks, impacting on various malignant cell functions such as proliferation, motility, angiogenesis and apoptosis [27-29]. HER2 amplification and/or overexpression have been detected in approximately 20% to 30% of patients with breast and gastric cancer and correlates with poorer clinical outcomes [19,30,31]. The importance of HER2 has been well documented in breast cancer, where HER2 testing is a standard approach for identifying patients who may benefit from HER2-targeted agents such as lapatinib and trastuzumab therapy in metastatic and adjuvant settings [32,33]. In gastric cancer, HER2 overexpression is associated with more aggressive disease and poor survival. Preclinical studies have indicated that trastuzumab can impede HER2-overexpressing human gastric cancer cells growth and inhibit tumorigenesis in xenograft models [34-36]. Accumulating studies indicate that HER2 overexpression may not be affected by gene amplification alone, but is also likely to be influenced by transcriptional activation and/or post-transcriptional mechanisms in cancers [28,37]. In previous reports, HER2 mRNA and protein overexpression have been directly affected by miRNA-mediated post-transcriptional mechanisms in carcinomas [38,39]. Our study also confirms that HER2 is a direct target of miR-331-3p. Considering the interaction of HOTAIR/miR-331-3p, we therefore hypothesize that HOTAIR may also regulate HER2 expression in gastric cancer, which signifies the role of HOTAIR in the tumorigenesis-regulating network.

In this study, luciferase and RIP assays confirmed the existence of specific crosstalk between the lncRNA HOTAIR and HER2 mRNA through competition for miR-331-3p binding. Consistent with HOTAIR sequestration of miR-331-3p, we found that its depletion reduced the expression level of HER2, while its overexpression restored elevated HER2 protein synthesis. These data are consistent with the hypothesis that ceRNAs are transmodulators of gene expression through competing miRNA binding. Furthermore, IHC and qRT-PCR assays revealed that HER2 was mainly upregulated in advanced stage gastric cancer tissues or those with lymph node metastasis, and associated with high HOTAIR expression. Altogether, the positive correlation between HOTAIR and HER2 expression and the relevance to miRNA expression levels (miR-331-3p/miR-124) supports our hypothesis that ceRNA can sequester miRNAs, thereby protecting their target RNAs from repression.

Lastly, the findings presented in this study have allowed us to conclude that HOTAIR overexpression represents an excellent biomarker of poor prognosis in gastric cancer, and may confer multiple properties required for tumor progression and metastatic phenotype. More importantly, our study indicates that the ceRNA activity of HOTAIR imparts a miRNA/lncRNA trans-regulatory function to protein-coding mRNAs and the ceRNA network may play an important role in gastric pathogenesis. Finally, our experimental data suggest that targeting the HOTAIR/HER2 interaction may represent a novel therapeutic application, thus contributing to better knowledge of the efficacy and tolerance of trastuzumab-based therapy in HER2-positive gastric cancer patients.

It is worth mentioning that the ceRNA activity of HOTAIR may sequester a handful of miRNAs at once, while one miRNA is also capable of controlling multiple genes. Therefore, the multiple properties of HOTAIR are likely due to simultaneous targeting of multiple targets in gastric cancer. We also hypothesize that there may be many other lncRNAs that function as ceRNAs to regulate expression of key genes in gastric cancer. Thus, the identification of these ceRNAs will undoubtedly enhance our knowledge of how lncRNAs function, allowing us to better understand the pathogenesis and development of gastric cancer and ultimately facilitate the development of lncRNA-directed diagnostics and therapeutics against this deadly disease.

## Materials and methods

### Tissue collection

Fresh-frozen and paraffin-embedded gastric cancer tissues and corresponding adjacent non-tumorous gastric samples were obtained from Chinese patients at Jiangsu province hospital between 2006 and 2008. All cases were reviewed by pathologist and histologically confirmed as gastric cancer (stageII,III,IV; 7th Edition AJCC) based on histopathological evaluation. Clinical pathology information was available for all samples (Table 1). No local or systemic treatment was conducted in these patients before the operation. The study was approved by the Research Ethics Committee of Nanjing Medical University, China. Informed consents were obtained from all patients.

### Cell lines and culture conditions

Four gastric cancer cell lines (MGC-803, SGC-7901, BGC-823, and AGS), a normal gastric epithelium cell line (GES-1), a NSCLC cell line (SPC-A1), a normal human bronchial epithelial cell line (16HBE), two breast cancer cell lines (MCF-7, MDA-MB-231), and a human embryonic kidney cell line (HEK293T) were purchased from the Institute of Biochemistry and Cell Biology of the Chinese Academy of Sciences (Shanghai, China). Cells were cultured in RPMI 1640 or DMEM (GIBCO-BRL) medium supplemented with 10% fetal bovine serum (10% FBS), 100 U/ml penicillin, and 100 mg/ml streptomycin (Invitrogen) in humidified air at 37 °C with 5% CO2.

### RNA extraction and qRT-PCR analyses

Total RNA was extracted from tissues or cultured cells using TRIZOL reagent (Invitrogen, Carlsbad, Calif). For qRT-PCR, RNA was reverse transcribed to cDNA by using a Reverse Transcription Kit (Takara, Dalian, China). Real-time PCR analyses were performed with Power SYBR Green (Takara, Dalian China). Results were normalized to the expression of GAPDH. For miR-331-3p and miR-124 expression detection, reverse transcription was performed following Applied Biosystems TaqMan MicroRNA Assay protocol (Cat. # 4427975 and Cat. # 4427975). U6 snoRNA was validated as the normalizer. The primers were listed in Additional file 3: Table S4. qRT-PCR and data collection were performed on ABI 7500.

### Plasmid constructs

HOTAIR cDNA was cloned into the mammalian expression vector pcDNA3.1 (Invitrogen). To express miRNAs, human microRNA precursors with about 80 bp of flanking sequences in both sides were amplified and cloned into the modified pLL3.7 vector (Invitrogen). To construct luciferase reporter vectors, HER2 3’-UTR and HOTAIR cDNA fragment containing the predicted potential microRNAs binding sites were amplified by PCR, and then subcloned downstream of the luciferase gene in the pLUC luciferase vector (Ambion, Inc.,Austin, TX, USA). Primers for subcloning and plasmid construction were listed in Additional file 4: Table S3. We also designed shRNA sequence targeted HOTAIR as shown in Additional file 3: Table S4. After annealing of the complementary shRNA oligonucleotides, we ligated the annealed oligonucleotides into pENTR vector (sh-HOTAIR).

### Transfection of gastric cancer cells

All plasmid vectors for transfection were extracted by DNA Midiprep kit (Qiagen, Hilden, Germany). Three individual HOTAIR siRNAs (si-HOTAIR) and scrambled negative control siRNA (si-NC) were purchased from Invitrogen (Invitrogen, CA, USA). Target sequences for HOTAIR siRNAs were listed in Additional file 3: Table S4. The si-HOTAIR, miR-331-3p or miR-124 was transfected into BGC-823 cells respectively, and pCDNA/HOTAIR was transfected into SGC-7901 cells using Lipofectamine2000 (Invitrogen) according to the manufacturer’s instructions. At 48 h after transfection, cells were harvested for qRT-PCR analyses or western blot.

### Cell proliferation assays

A cell proliferation assay was performed with MTT kit (Sigma, St. Louis, Mo) according to the manufacturer’s instruction. Cells were placed into 6-well plate and maintained in media containing 10% FBS for 2 weeks. Colonies were fixed with methanol and stained with 0.1% crystal violet (Sigma, St. Louis, Mo). Visible colonies were manually counted.

### Flow-cytometric analysis of apoptosis

BGC-823 cells transiently transfected with si-NC or si-HOTAIR were harvested 48 h after transfection by trypsinization. After the double staining with FITC-Annexin V and Propidium iodide (PI), the cells were analyzed with a flow cytometry (FACScan®; BD Biosciences) equipped with a CellQuest software (BD Biosciences).

### Hoechst staining assay

BGC-823 cells transiently transfected with si-NC or si-HOTAIR were cultured in six-well cell culture plates, and Hoechst 33342 (Sigma, St Louis, MO, USA) was added to the culture medium; changes in nuclear morphology were detected by fluorescence microscopy using a filter for Hoechst 33342 (365 nm). For quantification of Hoechst 33342 staining, the percentage of Hoechst-positive nuclei per optical field (at least 50 fields) was counted.

### Cell migration and invasion assays

At 48 h after transfection, cells in serum-free media were placed into the upper chamber of an insert for migration assays (8-μm pore size, millepore) and invasion assays with Matrigel (Sigma-Aldrich, USA). Media containing 10% FBS was added to the lower chamber. After several hours of incubation, the cells that had migrated or invaded through the membrane were stained with methanol and 0.1% crystal violet, imaged, and counted using an IX71 inverted microscope (Olympus, Tokyo, Japan).

### Tumor formation assay in a nude mouse model

5-week-old female athymic BALB/c mice were purchased from the Model Animal Research Center of Nanjing University. All animal procedures were performed in accordance to the protocols approved by the Institutional Animal Care and Use Committee at the Nanjing Medical University. For xenograft models, 5 × 10^6^ BGC-823 cells transfected with sh-HOTAIR and pENTR vector (EV) were injected subcutaneously in the right flank of BALB/c nude mice (five mice per group). Tumor volumes were examined every 3 days when the implantations were starting to grow bigger. After 16 days, these mice were sacrificed and tumors were weighted. Tumor volumes were calculated by using the equation V (mm^3^) = A × B^2^/2, where A is the largest diameter, and B is the perpendicular diameter. The primary tumors were excised and tumor tissues were used to perform qRT-PCR analysis of HOTAIR levels and immunostaining analysis of proliferating cell nuclear antigen (PCNA) protein expression.

### Bioinformatics methods

The potential microRNAs binding sites of HOTAIR predicted by computer-aided algorithms were obtained from Segal Lab (http://132.77.150.113/pubs/mir07/mir07_prediction.html), RegRNA (http://regrna.mbc.nctu.edu.tw/html/prediction.html) and microRNA.org-target program (http://www.microRNA.org).

### Luciferase assay

Human HEK293T cells (2.0 × 10^4^) grown in a 96-well plate were co-transfected with 150 ng of either empty vector or miR-331-3p, miR-124, 50 ng of firefly luciferase reporter comprising 3’UTR of HER2, wild type or mutant HOTAIR fragment, and 2 ng of pRL-TK (Promega, Madison, WI, USA) using Lipofectamie 2000 (Invitrogen, USA). rno-miRNA-344 acts as a negative control. Cells were harvested 48 h after transfection for luciferase assay using a luciferase assay kit (Promega) according to the manufacturer’s protocol. Transfection was repeated in triplicate.

### RNA Binding Protein Immunoprecipitation (RIP) assay

RNA immunoprecipitation was performed using the EZ-Magna RIP kit (Millipore, Billerica, MA, USA) following the manufacturer’s protocol. BGC-823 cells at 80-90% confluency were scraped off, then lysed in complete RIP lysis buffer, after which 100 μl of whole cell extract was incubated with RIP buffer containing magnetic beads conjugated with human anti-Ago2 antibody (Millipore), negative control normal mouse IgG (Millipore). Anti-SNRNP70 (Millipore)was used as positive control for the RIP procedure. Samples were incubated with Proteinase K with shaking to digest the protein and then immunoprecipitated RNA was isolated. The RNA concentration was measured using a NanoDrop (Thermo Scientific) and the RNA quality assessed using a bioanalyser (Agilent, Santa Clara, CA, USA). Furthermore, purified RNA was subjected to qRT-PCR analysis to demonstrate the presence of the binding targets using respective primers.

### Western blot assay and antibodies

Cells protein lysates were separated by 10% SDS-polyacrylamide gel electrophoresis (SDS-PAGE), transferred to 0.22 μm NC membranes (Sigma) and incubated with specific antibodies. Autoradiograms were quantified by densitometry (Quantity One software; Bio-Rad). GAPDH antibody was used as control, anti-HER2 (1:1000) and cleaved caspase-3 (1:1,000) were purchased from Cell Signaling Technology, Inc (CST).

### Immunohistochemistory (IHC)

Paraffin-embedded, formalin-fixed tissues were immunostained for HER2 and PCNA proteins. The signal was amplified and visualized with diaminobenzidine-chromogen, followed by counterstaining with hematoxylin. For HER2, an IHC score of 2+ or more was defined as positive, and IHC scores of 0 and 1+ were defined as negative [40]. Anti-HER2 (1:50) was purchased from Cell Signaling Technology, Inc. (CST), and anti-PCNA (1:50) was purchased from Bioworld Technology, Inc., respectively.

### Statistical analysis

Student’s *t*-test (two-tailed), One-way ANOVA and Mann–Whitney test were performed to analyze the in vitro and in vivo data using SPSS 16.0 software. P values less than 0.05 were considered significantly.

## Competing interests

All the authors hereby declare that they do not have any competing interests with regard to the manuscript submitted here for review.

## Authors’ contributions

XHL, MS, KMW carried out the molecular genetic studies, participated in the sequence alignment and drafted the manuscript. YBG carried out the immunoassays. FQN, EBZ, DDY, RK, KHL, XFC, JHL participated in the design of the study and performed the statistical analysis. WD, ZXW conceived of the study, and participated in its design and coordination and helped to draft the manuscript. All authors read and approved the final manuscript.

## Supplementary Material

Additional file 1: Figure S1 **(A)**. MTT assay was performed to determine the proliferation of pCDNA/HOTAIR transfected MGC803 and AGS cells. Data represent the mean ± s.d. from three independent experiments. **(B)**. Western blot analysis of cleaved caspase-3 after si-HOTAIR transfection with BGC-823 cells. GAPDH was used as a control. *P < 0.05. Click here for file

Additional file 2: Table S2 Immunostaining of HER2 protein in advanced GC tissue samples. Click here for file

Additional file 3: Table S4 Primers for qRT-PCR analyses and target sequences for HOTAIR siRNAs or shRNA. Click here for file

Additional file 4: Table S3 Primers for subcloning and plasmid construction. Click here for file
