# Supplementary figures and images for: Lnc RNA HOTAIR functions as a competing endogenous RNA to regulate HER2 expression by sponging miR-331-3p in gastric cancer
Source: Mol Cancer. 2014 Apr 28;13:92. doi: 10.1186/1476-4598-13-92 (PMC4021402; doi:10.1186/1476-4598-13-92)

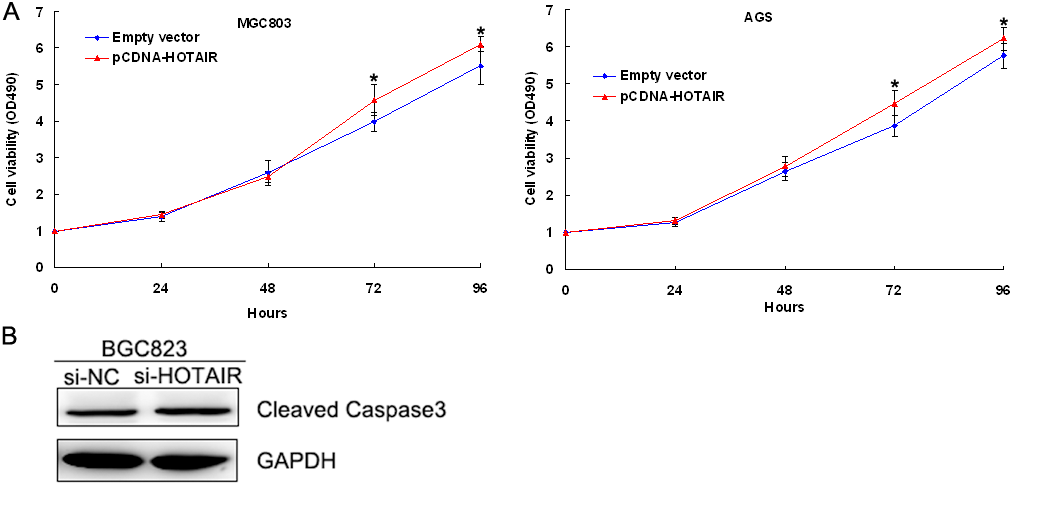

Supplement: Additional file 1: Figure S1 — (A). MTT assay was performed to determine the proliferation of pCDNA/HOTAIR transfected MGC803 and AGS cells. Data represent the mean ± s.d. from three independent experiments. (B). Western blot analysis of cleaved caspase-3 after si-HOTAIR transfection with BGC-823 cells. GAPDH was used as a control. *P < 0.05. [file 1476-4598-13-92-S1.tiff]
