# Supplementary material for: Lnc RNA HOTAIR functions as a competing endogenous RNA to regulate HER2 expression by sponging miR-331-3p in gastric cancer
Source: Mol Cancer. 2014 Apr 28;13:92. doi: 10.1186/1476-4598-13-92 (PMC4021402; doi:10.1186/1476-4598-13-92)
Supplement: Additional file 2: Table S2 — Immunostaining of HER2 protein in advanced GC tissue samples. [file 1476-4598-13-92-S2.doc]

|  | Relative low HOTAIR expression  (n=25) | | Relative high HOTAIR expression  (n=25) | |
| --- | --- | --- | --- | --- |
|  | stage  Ⅲa/Ⅲb | stage  Ⅲc/Ⅳ | stage  Ⅲa/Ⅲb | stage  Ⅲc/Ⅳ |
| HER2 negative  immunostaining | 15(60%) | 2(8%) | 3 (12%) | 2 (8%) |
| HER2 positive  immunostaining | 2(8%) | 6(24%) | 2 (8%) | 18(72%) |

**Additional file 2: Table S2. Immunostaining of HER2 protein in advanced GC tissue samples**

The 50 gastric cancer patients were classified into two groups: High-HOTAIR group (n=25): HOTAIR expression ratio ≥ median ratio; Low-HOTAIR group (n=25): HOTAIR expression ratio ≤ median ratio, and were immunostained for HER2.
