# Supplementary material for: Lnc RNA HOTAIR functions as a competing endogenous RNA to regulate HER2 expression by sponging miR-331-3p in gastric cancer
Source: Mol Cancer. 2014 Apr 28;13:92. doi: 10.1186/1476-4598-13-92 (PMC4021402; doi:10.1186/1476-4598-13-92)
Supplement: Additional file 3: Table S4 — Primers for qRT-PCR analyses and target sequences for HOTAIR siRNAs or shRNA. [file 1476-4598-13-92-S3.doc]

**Additional file 3: Table S4. Primers for qRT-PCR analyses and target sequences for HOTAIR siRNAs or shRNA**

| HOTAIR forward: | 5’-CAGTGGGGAACTCTGACTCG-3’ |
| --- | --- |
| HOTAIR reverse: | 5’-GTGCCTGGTGCTCTCTTACC-3’ |
| HER2 forward: | 5’-TGACACCTAGCGGAGCGAT-3’ |
| HER2 reverse: | 5’-GGGGGATGTGTTTTCCCTCAA-3’ |
| GAPDH forward: | 5’-GGGAGCCAAAAGGGTCAT-3’ |
| GAPDH reverse: | 5’-GAGTCCTTCCACG ATACCAA-3’ |
| U6 forward: | 5’-CTCGCTTCGGCAGCACA-3’ |
| U6 reverse: | 5’-AACGCTTCACGAATTTGCGT-3’. |
| si-HOTAIR1: | 5’-AAAUCCAGAACCCUCUGACAUUUGC-3’ |
| si-HOTAIR2: | 5’-UUAAGUCUAGGAAUCAGCACGAAGC-3’ |
| si-HOTAIR3: | 5’-CAUAUUAUAGAGUUGCUCUGUGCUG-3’ |

sh-HOTAIR sense: 5’-CACCGCCTTTGCTTCGTGCTGATTCCGA

AGAATCAGCACGAAGCAAAGGC-3’

sh-HOTAIR anti-sense: 5’-AAAAGCCTTTGCTTCGTGCTGATTCTTCGG

AATCAGCACACGAAGCAAAGGC-3’
