# Supplementary material for: Lnc RNA HOTAIR functions as a competing endogenous RNA to regulate HER2 expression by sponging miR-331-3p in gastric cancer
Source: Mol Cancer. 2014 Apr 28;13:92. doi: 10.1186/1476-4598-13-92 (PMC4021402; doi:10.1186/1476-4598-13-92)
Supplement: Additional file 4: Table S3 — Primers for subcloning and plasmid construction. [file 1476-4598-13-92-S4.doc]

**Additional file 4: Table S3. Primers for subcloning and plasmid construction**

| pCDNA3.1-HOTAIR forward: | 5’-CATGGATCCACATTCTGCCCTGATTTCCGGAACC-3’ |
| --- | --- |
| pCDNA3.1-HOTAIR reverse: | 5’-ACTCTCGAGCCACCACACACACACAACCTACAC-3’ |
| pLL3.7-pre-miR-331-3p forward: | 5’-CACAACTCGAGAACGTACAGAAGGCTCCAGAAATG-3’ |
| pLL3.7-pre-miR-331-3p reverse: | 5’-TGAAGATCTGAAGGATTAACCAACCAATTTTTGC-3’ |
| pLL3.7-pre-miR-124 forward: | 5’-CACAAGTCGACGTCTACACTTCCACGGAACAGACT-3’ |
| pLL3.7-pre-miR-124 reverse: | 5’-AAGGATCCCACTAGGTTGCTTCCAGAATCAGT-3’ |
| pLUC-HOTAIR forward: | 5’-CACAACTCGAGGTGAAAGCGAACCACGCAGAGAAA-3’ |
| pLUC-HOTAIR reverse: | 5’-TGAAGATCTAGCTACATGTGAGTATATACTCCATA-3’ |
| pLUC-HER2-3'UTR forward: | 5’-ACCAGAAGGCCAAGTCCGCAGAAG-3’ |
| pLUC-HER2-3'UTR reverse: | 5’-TAGCTGTTTTCCAAAATATATTTGC-3’ |
